# The key enablers of competitive advantage formation in small and medium enterprises: The case of the Ha’il region

**DOI:** 10.3389/fpsyg.2022.1030405

**Published:** 2022-10-28

**Authors:** Murad Thomran, Mohammad Alshallaqi, Yaser Hasan Al-Mamary, Mohammed Abdulrab

**Affiliations:** ^1^Department of Accounting, College of Business Administration, University of Hail, Hail, Saudi Arabia; ^2^Department of Management and Information Systems, College of Business Administration, University of Hail, Hail, Saudi Arabia; ^3^Management Department, Community College of Qatar, Doha, Qatar

**Keywords:** competitive advantage, SMEs, entrepreneurial orientation, Ha’il region, entrepreneurship

## Abstract

The primary objective of this research is to establish the extent to which small and medium-sized businesses (SMEs) in the Ha’il region benefit from a significant competitive advantage brought about by an entrepreneurial mindset (innovativeness, proactiveness, risk-taking, competitive aggressiveness, and autonomy). To achieve these objectives, the study used a questionnaire to collect data. A total of 220 SMEs in the Ha’il region were surveyed. The participants completed an online self-administered survey and used the PLS-SEM technique. The researchers found a robust link between differentiation advantage and higher levels of innovativeness, proactiveness, risk-taking, competitive aggression, and autonomy. In addition, the outcomes of the survey reveal that a greater cost advantage is substantially associated with vastly greater innovativeness, proactiveness, risk-taking, and competitive aggression overall. However, cost advantage is not strongly correlated with autonomy. These findings are significant because they shed new light on how competitive advantages are formed through the entrepreneurial orientation of entrepreneurs in the Ha’il region. This is a significant theoretical contribution to the literature on entrepreneurial orientation, specifically in the context of SMEs. The findings may also be valuable in supporting SMEs in being successful by enhancing their competitiveness, as SMEs are key contributors to the development and growth of the economy.

## Introduction

The idea of a competitive advantage refers to a group of characteristics or competencies that give a company an advantage over its competitors in terms of its ability to consistently generate higher profits (Roberts, 2002; Dagnino et al., 2021). A competitive advantage might stem from a firm’s ability to lower its costs significantly below those of its competitors, thus enjoying higher profit margins, or from developing high-end niche products and services that are difficult to find somewhere else (Thomson and Strickland, 2002; Huang et al., 2015). According to Barney (2001), a company is said to have a competitive edge when it is able to create value in ways that are either distinctive or more cost-effective than those of its competitors. In addition, for a competitive advantage to be of any use, customers’ perceptions of the company must be positive, and they must perceive the company’s identity differently from that of its competitors. Porter (1987) revealed that the major factors of competitive advantage are technical innovation, professional reputation, and healthy organizational relationships. Hitt et al. (1997) stated that each firm is a collection of diverse resources and talents, beginning with a resource-based perspective of the company. These available resources and creative skills make it possible to build and maintain a competitive advantage (see also Zahra, 2021).

Such resources and capabilities drive innovation, reputation, and relationships and help a firm capitalize on its core competencies (Gibson et al., 2021; Hossain et al., 2021). Core competencies can be seen as niche areas of expertise that stem from the intermingling of technological systems, structures, and work practices (Prahalad and Hamel, 1990; Jalil et al., 2021). Prahalad and Hamel (1990) argued that the following three key attributes characterize core competencies: (1) a competitive core competency enables access to different markets; (2) competitive core competencies enhance consumer perception of the benefits they may accrue from the use of a firm’s products and/or resources; (3) core competitive competencies are difficult for competitors to replicate. Therefore, to build a competitive advantage successfully, a firm must offer what consumers perceive as superior value.

A sustainable competitive advantage is usually attained when firms generate exceptional value through capitalizing on their unique mix of resources, capabilities, and core competencies (Hitt et al., 1997; Davis and DeWitt, 2021). This assertion stems from the resource-based view, which argues that unique resources and capabilities are the key drivers of competitive advantage (Barney, 2021). For example, a firm can develop a strong competitive advantage if it has strong capabilities and resources in research and product development. Firms with strong research capabilities are typically pioneers in their industries and can, therefore, sustain their competitiveness for extended periods (McGee et al., 1999; Lu et al., 2021).

Network effects are another source that can be harnessed to develop a competitive advantage. For example, if a firm’s offerings are of greater quality, then consumers are typically attracted to that firm and inclined to use its offerings. When Microsoft developed its user-friendly Windows operating system, it was easier than the older DOS system because it had a user interface. People, therefore, liked it and started recommending it through word-of-mouth to people in their networks. Those people then did the same and spread the word through their networks. This is an example of how network effects can also be used to sustain a competitive advantage (McIntyre and Srinivasan, 2017).

The number of small and medium-sized businesses (SMEs) has grown tremendously in the Ha’il region because of economic development plans, such as Vision 2030, and prior economic development schemes that aim to enhance the competitiveness of every region in Saudi Arabia. The Ha’il region has the potential to build an entrepreneurial environment to attract SMEs and help them grow. Activities have taken place between the SME authority in Saudi Arabia (Monsha’at) and the Commission for the Development of the Ha’il region, as well as the University of Ha’il. Therefore, more needs to be researched and understood regarding the mix of resources, capabilities, and core competencies in the Ha’il region that can be utilized to build an entrepreneurial environment conducive to SMEs’ competitiveness.

An enterprise’s competitiveness is its ability to outperform the competition in terms of revenue generated. There are two sources of an organization’s competitiveness: differentiation in terms of niche markets or superior quality offerings, as well as lower cost, which stems from economies of scale. For a competitive advantage to be sustainable over the long term, its sources must be unique and difficult to replicate (Al-Mamary et al., 2022).

This research project aims to help these efforts by enhancing the current understanding of the different factors that influence the competitiveness of SMEs in the Ha’il region and of what can be done to build a unique entrepreneurial environment in the Ha’il region. This project will focus on entrepreneurial orientations in the Ha’il region that can be harnessed to help SMEs develop and sustain a competitive advantage. This research project will conduct a thorough study of the dimensions of entrepreneurial orientations in the Ha’il region that might shape the formation and endurance of an organization’s competitive advantage. The focus will be on SMEs, given that one of the priorities of economic development in this region is to foster an entrepreneurial environment that promotes the development of SMEs.

## Literature review

### Strategic competitive advantage general overview

Sultan and Mason (2010) developed the basic principle of competitive advantage throughout SMEs in developing countries by stating that the economic viability of a company or organization is best attained through a competitive advantage; thus, when developing business plans, it is essential to meet customers’ needs, as well as increase the level of customer satisfaction. These and other beliefs seem to be the basis for a new competitive business model, in which new products and services offered to the market are presented to existing and new customers at affordable and reasonable rates. This is done in the components of the marketing segmentation or in improved attention to specific customers’ requirements in a highly specialized segmented market compared to industry rivals in a relatively similar business sector.

Competitive advantage has always been defined as an institution’s potential capacity to distinguish its products or services from those of its competing industry rivals. Furthermore, a competitive advantage is necessary to create an effective business strategy aimed at achieving protected economic growth (Simpson et al., 2004). Jones (2003) developed the key characteristics of competitive advantage, particularly with regard to the formation of value propositions, and indicated the following three generic competitive strategies: the basic cost leadership process, long-term product or service differentiation, and focusing on products or services. These and other key marketing techniques can quickly contribute to the success of business goals and are widely used among different SMEs.

Thus, to outperform competitors, business organizations must promote additional economic value for their own products and services to their own customers (Barney and Hesterly, 2010). Consequentially, to gain a competitive advantage that encompasses the entire business process, a business could emphasize the fundamental value systems specific to its customer base. Users should be able to distinguish a company’s goods and/or services from those of its industry rivals even after they have fully recognized the basic value systems of these kinds of goods and services. Nevertheless, in the particular instance of young entrepreneurs facing intense rivalries in a competitive market environment, achieving a competitive advantage depends on the corporate business environment.

In fact, there are three different elements of competitive advantage: (1) efficient goods and services at cost leadership, (2) fully branded, differentiated company goods or services, and (3) the product or service responds to specific customers’ needs in a particular geographical location in terms of target market segmentation. The competitive advantage strategies chosen for SMEs and new entrepreneurs must be highly adaptable because they are heavily reliant on market trends, industry structure, and environmental forces that promote the emergence of significant strategic competitive advantages (Gassmann and Keupp, 2007). In this particular respect, new opportunities and enterprises must devote their own resources, business knowhow, and business capabilities to an efficient collaborative effort with suppliers’ wider distribution channels and middleman channel partners to gain a competitive advantage throughout all value chain business processes (Pavic et al., 2007). These and other components contribute to a company’s overall success in achieving a competitive advantage, which in turn could be used to develop an effective company strategy for long-term development and sustainability.

Each organization’s potential to maintain a competitive advantage tends to vary with the corporate business environment, regardless of whether advanced technologies or interorganizational collaborative efforts are used to gather information. Zaridis (2009) stated that competitive advantage is important for young entrepreneurs because it enables a business to achieve sustainable development, as well as defensive capabilities, as a prerequisite for the successful monitoring of human and financial resource management. Hence, startups should closely examine all the various internal and external business macroenvironmental contextual factors. Similarly, working to develop a significant competitive advantage through competitive cost leadership, innovation, and business differentiation is critical, as is the ability to respond to the requirements of a given segment of individuals in full compliance with both the employment options and challenges of the institution’s surrounding social and physical environment. Such a practice is consistent with the school of thought of suboptimal resource utilization for differentiated product market strategy. Furthermore, this practice assists a business enterprise in clearly distinguishing itself from its own industry rivals and prevents potential challenges and barriers to product substitution.

### Dimensions of competitive advantage

The 17 According to Porter (1980, 1985), a company’s product differentiation and cost leadership were indeed the only two generic strategies formulated to achieve a key market competitive advantage for an entire organization. Retail business customers appreciate product differentiation, which many perceive as a new technique because it satisfies the customers’ basic demands. Conversely, cost leadership emphasizes reasonably low product costs in comparison to industry rivals (Porter, 1980, 1985). Porter (1980, 1985) further argued that cost leadership and product differentiation strategies have always been mutually incompatible. However, popular literature reviews and other similar scientific papers have challenged this mistaken notion, acknowledging that business organizations might consider pursuing components of both types of market strategy (Chenhall and Langfield-Smith, 1998).

Furthermore, a business organization has a competitive advantage when it can provide relatively similar economic advantages of competing companies at a relatively lower cost or when it can provide economic benefits that exceed the normal benefits or satisfaction given to customers. Therefore, the basic component of a competitive edge can be something that the business organization does that is completely unique, new, or extremely difficult to replicate (Prahalad and Hamel, 1990).

Essentially, competitive advantage must be created and preserved while satisfying customer requirements (Prahalad and Hamel, 1990; Ozbekler and Ozturkoglu, 2020). Conversely, cost leadership helps to create a significant value system, meaning providing excellent goods or services at a significantly lower cost than industry rivals or offering differentiation, i.e., delivering goods or services generally perceived to be exceptionally unique in relation to a certain essential feature (Markides and Williamson, 1994). Acknowledging how much each competitive level’s pertinent resource base and technological capability influences costs and uniqueness is crucial in determining whether each must add value to the products and services made available (Duncan et al., 1998; Fainshmidt et al., 2019).

#### Cost as a dimension of competitors in the market

The primary emphasis on competitive cost discounts is perhaps the most important factor in predicting commonly used components by organizations, particularly those in markets where employees and customers appear to be responsive to price changes. Key factors that contribute to cheaper prices include comprehensive guides, academic credentials, professional training, fruitful expenditure, the implementation of appropriate manufacturing, and dissemination of policy initiatives (Deborah, 1998; Leiblein et al., 2017). Today, enterprises with this particular dimension frequently have monopolistic tendencies and the ability and willingness to develop a competitive advantage. Furthermore, organizations achieve this competitive advantage because their aggregated costs for economic activities are lower than those of competing companies.

#### Differentiation as a competitive dimension

Organizations perceive product differentiation as a far more essential element and distinguishable way of accomplishing a competitive advantage than that of a low-cost product business strategy (Kotha and Orne, 1989; Baines and Langfield-Smith, 2003). According to Dirisu et al. (2013) and Barney (1991), a business organization has a competitive advantage because once this is put in place, it creates a value creation strategy plan that current or future potential industry rivals do not have. Furthermore, competitive advantage can be defined as a company’s current business advantage over existing rival companies.

#### Competitive dimensions of increased flexibility

The business organization’s innate ability and financial power to provide the same wide range of important distinctions, as well as changes in the level of the customer base, result from its willingness to manage technological advancements. Instead, they design goods and services based on consumer expectations (Oberholzer-Gee and Yao, 2018). Organizations must react quickly to changes in consumer preferences, whether they rise or fall, and this is an essential component for competitive reasons because it allows for quickly serving customers’ basic needs.

According to Karajewski and Ritzman (2005), flexibility is a corporate business operation that allows for efficiently meeting the basic needs of customers. Dillworth (1996) stated that flexibility, the ability to adjust and respond to consumers’ needs and prevent unnecessary customer grievances, enables excellent customer service. Furthermore, to minimize overarching costs, business organizations control a larger market ownership stake than most other industry rivals.

#### Delivery as a competitive dimension

A business consumer’s motivation is the willingness to pay a comparatively high price for the products or services they typically use in a given timeframe (Al-Bakri, 2005). Business organizations appear to be dynamic and responsive to consumers’ basic essentials and desires when they obtain more customers willing to pay exorbitant prices for goods and services, at least until the major competing companies decide to enter the consumer-based retail market. According to Noori and Radford (1995), business organizations are likely to maintain competitive advantages placed above and beyond their industry rivals once costs are minimized and a substantial market share is achieved. Effective customer service delivery can be characterized as receiving a customer’s requirements and then satisfying them within a given timeframe (Martins, 2020).

#### Quality as a competitive dimension

Business organizations that provide goods and services have always been concerned with the perceived value of those goods and services, which would, in turn, manage to achieve some level of service quality and reasonable and fair customer demand expectations through the visual structural design of the goods and services, particularly in terms of the perceived value of the customer’s new company’s products (Al-Bakri, 2005; Parker et al., 2017). Numerous business organizations strive for continuous improvement in the overall quality of their goods or services to compete with competitors. Overall, service quality as a competitive tool requires organizations to view service quality as a means of satisfying their customer base rather than as a means of solving structural problems and keeping costs low (Baker, 1992). A certain business organization can achieve a larger presence, a significantly higher rate of profitability, a higher sense of fulfillment to properly manage market value prices, and significant increases for services performed while also providing excellent goods or services.

According to Porter (1980, 1985) and Al-Mamary et al. (2020a), differentiation and cost advantage were the two main generic strategies to achieve a key market competitive advantage; hence, this study will focus on these two dimensions.

### Entrepreneurial orientation model

#### Competitive advantage and innovativeness

Innovativeness is a company’s proclivity to foster the development of truly innovative concepts, integrate advanced technologies, and move ahead with current product lines or service offers. Amodu and Aka (2017) and Edwards et al. (2014) described innovativeness as the propensity to pursue creative thinking and experiment with new ideas. Innovations result in enhanced skills and techniques for achieving full incremental improvements, while radical incremental innovations necessitate the acquisition of completely new skills and might even render current talent largely redundant. In any particular instance, the primary objective of organizational creativity is to create new products, services, systems, and processes. Major successful business organizations that have achieved enormous success in their organizational innovation outperform their competitors.

Innovativeness reflects a company’s natural propensity to pursue and continue to support new innovative thoughts, uniqueness of ideas, research, and experimentation, which might also lead to more efficient offerings or technological improvements (Lumpkin and Dess, 1996a; Al-Mamary et al., 2020b). The different goods and services that the business organization has launched in the real market are referred to as its innovativeness. According to some scientific theorists, innovation is intimately connected to entrepreneurial behavior because small business owners generate new and improved combinations of resource management simply by entering a new marketplace. In the specific situation of entrepreneurial orientation (EO), innovativeness is described as a more narrowly focused term, emphasizing the business’s organizational meaning and significant market leadership, as well as the need for change in its core product offerings (Schillo, 2011). Hence, autonomy is considered an essential consideration of an EO mindset.

#### Competitive advantage and proactiveness

Significant risk-taking has always been defined as the proclivity to actively participate in extremely brave but conservative behavioral responses. Proactiveness, however, can be defined as a way for a business organization to perform its functions for enterprises in a complex, turbulent system or in early-stage areas of the economy where environmental circumstances are constantly changing and growth opportunities and chances for success abound. Proactivity is a forward-thinking, excellent opportunity mindset that entails implementing innovative goods and services better than competition and planning and preparing for a possible market to make a real change happen and maintain the environment (Lumpkin and Dess, 2001; Patel et al., 2014). Proactivity is the ability to prepare for and respond to long-term goals instead of responding appropriately to major events as they actually happen. A proactive, instead of reactive, organization is one that seeks new opportunities. Such business organizations act ahead of fluctuating business demand and therefore are frequently either the first ones to expand their business and bring in new customers or “fast followers,” who enhance the continued efforts of the first-moving companies (Edwards et al., 2014).

Lumpkin and Dess (1996a) described proactiveness as taking action in anticipation of possible negative issues. Astrini et al. (2020) described proactiveness as the capacity and willingness to formulate strategies based on economic opportunities newly discovered through independent research and predictive market trend analysis. Proactivity helps businesses gain a competitive advantage while also placing the market competition in a position where it must provide a general response to the first-movers’ new initiatives.

#### Competitive advantage and risk-taking

The propensity to actively participate in courageous rather than conservative behavioral actions is known as risk-taking (Edwards et al., 2014). Choosing to take risks has traditionally been strongly correlated with entrepreneurial behavior. Hence, risk-taking specifically refers to willingness to accept the potential consequences of something, such as when individuals work for themselves instead of being gainfully employed by someone else or when senior management makes a decision to dedicate considerable resources to major projects with unpredictable consequences (Schillo, 2011; Stagni et al., 2021).

#### Competitive advantage and competitive aggressiveness

Competitive aggressiveness corresponds to whether businesses respond to existing market trends and new demands throughout the competitive global consumer market system. Competitive aggressiveness considers the concentration of a company’s business and continued attempts to outperform rival companies, as manifested by a confrontational position or an aggressive reaction (Lumpkin and Dess, 2001; Andrevski and Ferrier, 2019). Lumpkin and Dess (1996b) defined a competitive advantage of an aggressive nature as a company’s business proclivity to effectively and passionately overcome its own market competition to gain access or continue improving placement to outperform existing competitors in the global consumer market system. Business organizations demonstrate competitive aggressiveness when they vigorously pursue their competitors’ business opportunities (Schillo, 2011).

#### Competitive advantage and autonomy

The term “autonomy” refers to whether an individual or a group of individuals within an organization has the freedom to formulate and implement a start-up business idea. Individuals in a slightly elevated business organization have the freedom to hire those who introduce different concepts and are free from the straitjacket of bureaucratic inefficiency. Autonomy enables individuals and business organizations to more efficiently and successfully investigate and implement innovative thoughts without being constrained by organizational values and traditional practices (Edwards et al., 2014). From an EO business standpoint, autonomy exclusively focuses on system autonomy. Those same increased concentrations or business strategy measurements of autonomy enable a group of people (or ordinary people) to not only identify a problem but also target requirements for mitigating the occurrences of such a problem. Entrepreneurial autonomy usually involves having the innate potential to decide what, how, and when a private equity project could be accomplished and the business’s overall future plan (Lumpkin et al., 2009; Bledow et al., 2021). Furthermore, Lumpkin and Dess (1996b) revealed that autonomy refers to an individual’s or group of individuals’ progressive development in attempting to bring an undefined new concept or a sense of direction while successfully seeing said concept through its delivery stages. In a broad sense, autonomy refers to the ability and commitment to achieve potential business opportunities on one’s own. This then refers to the actions taken in the absence of a business organization’s resource constraints.

### The entrepreneurial mindset’s similarities and distinctions

The innovation metric is concerned with the introduction of innovative products and services, as well as the creation of enhanced versions of current products and services, as well as the introduction of unique techniques and procedures for the manufacture of those products. It was also stated that a firm’s tendency to be on the cutting edge of new technologies demonstrated that it had an entrepreneurial spirit if the organization tended to be at the forefront of such innovations (Antoncic and Antoncic, 2011).

In addition, “proactiveness” is defined as the degree to which a company strives to set the standard in critical business areas such as the introduction of new products or services, operating technology, and administrative practices, rather than merely following competitors in these areas (Antoncic and Hisrich, 2003). Proactiveness is measured as the percentage of a company’s overall efforts to do so. In other words, the degree to which a company goes above and beyond in its efforts to compete with others in its area is considered to be a measure of the organization’s proactiveness. Being proactive means making changes that shake up the way people think and giving assertive decision-making more weight than tactical approaches.

An entrepreneurial strategy that incorporates components of risk-taking and experimentation, such as a mindset that is bold, directive, and opportunity-seeking, is consistent with risk-taking in the same way (Antoncic and Antoncic, 2011). Taking calculated chances necessitates exhibiting initiative, competitive aggressiveness, and boldness in one’s views and actions, all of which are matched by the top management in the organization. These dimensions, which are related to entrepreneurship at the level of the firm, combine earlier categorizations. For instance, previous research has demonstrated that autonomous behavior, competitive aggression, and risk-taking all differ from one another in their own unique ways.

The evidence implies that risk-taking and competitive aggressiveness should share a component with proactiveness (Al-Mamary and Alshallaqi, 2022). Independence, which was previously thought of as a component of an entrepreneurial mindset but was found to emerge at the personal level rather than at the organizational level, was captured in this study with the addition of new dimensions. However, it was found that independence emerged at the personal level rather than at the organizational level.

The dimensions are distinct from one another in terms of the activities they engage in and the ways in which they handle different situations. The new business-venturing component of the company focuses on seeking and entering new companies within the existing organization that are relevant to the company’s current products or markets. When it comes to the innovativeness component, the primary focus is on the development of cutting-edge goods, processes, and technologies. This is because innovation is directly correlated to increased competitive advantage (Chahal and Bakshi, 2015). The self-renewal dimension places a significant emphasis on putting one’s attention toward the reformulation, reorganization, and transformation of organizational strategies. Satyanarayana et al. (2022) reported that the proactiveness statistic is a representation of the top management’s commitment to generating higher competitiveness. Thus, proactiveness comprises initiative, risk-taking, competitive aggression, and boldness. Because of this, it is feasible that every aspect of an entrepreneur’s personality can change in its own unique way. It is possible to break apart the numerous dimensions of intrapreneurship into their own distinct concepts.

This suggests that the basis of intrapreneurship can be found in the fact that various dimensions can be separate from one another while also being associated with one another. From these points of view, the concept of “intrapreneurship” is made up of these dimensions, which are different enough from each other to keep them from being duplicated but similar enough to be thought of as part of the same concept.

### Entrepreneurial personality traits

The personality aspects of entrepreneurship were previously used to characterize a person’s “big 5” personality traits have been renamed “OCEAN,” by (Antoncic et al., 2015) and the early taxonomy-building efforts can best be summarized as follows: -.

#### Openness

Because it makes it easier to find new opportunities for business, being open to new ideas is an essential quality for entrepreneurs to possess because it speeds up the process of finding new ventures to pursue (Antoncic et al., 2018; Awwad and Al-Aseer, 2021). Therefore, the ability to act quickly and decisively is critically important for success in entrepreneurship. Kritikos (2022), Tsaknis et al. (2022), and Postigo et al. (2021) studies that looked at the connection between personality and entrepreneurship found that how open someone is to new ideas is a big part of whether or not they will be successful as an entrepreneur.

A person who actively looks out for business opportunities and turns them into viable entrepreneurs is referred to as an “entrepreneur.” When beginning a business, one of the most critical skills to have is the ability to recognize opportunities ahead of time and capitalize on them before others do. Consequently, the first step toward being an entrepreneur is being able to recognize an opportunity.

#### Conscientiousness

Brockhaus Sr (1980) has shown that people who start their own businesses like to make choices that involve a moderate amount of risk, despise performing duties that are repetitive, and look for knowledge about the precise consequences of the decisions they make. Since the content of these traits is similar to that of the big five factors, it is clear that the drive to succeed is one of the most important parts of being conscientious.

Zhou et al. (2018) also observed that highly conscientious people are characteristic of the entrepreneurial-type, and among the big five personality qualities, conscientiousness has the strongest association with entrepreneurial status compared to managerial status. This is the case when comparing the five big personality traits to each other.

#### Extraversion

People who fall under the category of extraverts are typically self-assured and authoritative, in addition to being vivacious, daring, and exuberant. Because of this, it was discovered that people who owned their own businesses had a more positive attitude toward life than people who did not own their own businesses. Extroverts have a greater propensity to be upbeat, cheerful, and enthusiastic than introverts do.

In addition, Leutner et al. (2014) reported that entrepreneurs typically had high scores on the measures measuring conscientiousness and extraversion. Therefore, extraversion may be an asset to the success of an effective leader.

#### Agreeableness

The term “agreeability” refers to a broad variety of human attributes, any one of which may have a favorable or negative impact on one’s ability to run a successful business (Anitei, 2015). Entrepreneurs typically have a great deal of drive and ambition, but if they aren’t careful, this may work against them in a significant way, causing their firms and careers to suffer. Certain business entrepreneurs believe that the ways in which they conduct their operations are the only ones that should be followed.

As a result, those that venture out on their own in business face significant entrepreneurs while attempting to adapt. On the one hand, working with entrepreneurs may be motivating because of their unlimited excitement, charismatic personalities, strong competitiveness, and laser concentration on accomplishing their goals. This means that an individual’s unhealthy preoccupation with micromanaging every aspect of their business could have a detrimental impact on the quality of their personal relationships.

#### Neuroticism

The stereotype of entrepreneurs is that they are eccentric people whose behavior and ideas are shocking to others and go against the grain of society (Anitei, 2015). Because they are under a great deal of pressure, they are erratic and they make decisions too quickly for their own good. Because of this, we need to view the entrepreneur as a character with a great deal of dimensionality. There may be a consistent pattern of behavior among entrepreneurs that can be traced back to the problems they faced when they were just starting out.

Hence, entrepreneurs’ tendency toward impulsivity, unhappiness, rejection, or lack of control may undermine their confidence. When faced with these challenges, business owners and entrepreneurs may develop a fixation on opportunities to demonstrate their authority and autonomy, which may prevent them from meeting the requirements of individuals in their immediate environment. Therefore, the majority of the research that has been carried out up to this point reveals that neuroticism has a negative correlation with the ownership of an entrepreneurial enterprise.

### The direct correlation between entrepreneurial orientation and competitive advantages

Regarding strategy information (generic strategies) in conjunction with strategic planning, shaping the processes of entrepreneurial success appears to have become a rational and reasonable line of independent investigation. This orientation provides a framework for developing and incorporating competitive strategies. Thus, researching and developing the actual content of entrepreneurial orientation and competitive strategy is a potentially fruitful undertaking of research (Rauch et al., 2009; Wales et al., 2011; Lechner and Gudmundsson, 2014; Al-Harasi et al., 2021). According to Van Geenhuizen et al. (2008), entrepreneurial orientation has been identified as a potentially effective alternative to the negative issues that businesses and organizations face in achieving long-term, unique competitive advantages. Thus, there appears to be a significant concern in broadening SMEs’ understanding of the complexities of entrepreneurial orientation. Various facets of entrepreneurial orientation have different influences on competitive advantages (Lechner and Gudmundsson, 2014; Chen and Miller, 2015).

### Recent research findings on entrepreneurial orientation and competitive advantages from the perspective of Saudi Arabia

Alzahrani (2020) investigated the long-term impact of entrepreneurial orientation on organizational effectiveness and the influence of absorptive capacity. The results demonstrated that organizational entrepreneurial orientation had a substantial influence on business performance and that project management success mediates the whole correlation substantially. Furthermore, absorptive capacity moderated the correlation between entrepreneurial orientation and project management success, and this correlation became larger and more powerful with the mere existence of absorptive capacity.

Abdulrab et al. (2021) explored the influence of strategic orientations in mediating the correlation between entrepreneurial orientation and efficient implementation in Saudi SMEs. The research findings revealed that KSA SME management teams should maintain an intense focus on entrepreneurial behavior and develop a unique business strategy approach to achieve overall effectiveness. It was strongly suggested that decision makers demonstrate business and management initiatives to help SMEs shape entrepreneurial ventures.

Al-Mamary et al. (2020a) reviewed the existing literature on entrepreneurial orientation to establish the severity with which entrepreneurial orientation strongly influences the financial and nonfinancial achievements of Saudi SMEs. The research findings further clarified the correlation between such a company’s business entrepreneurial orientation and its financial and nonfinancial achievements.

Albasri (2020) considered the effect of entrepreneurial orientation combined with three main functionalities in helping to improve the efficiency of Saudi SMEs. According to the research results, entrepreneurial orientation, exploration, exploitation, and realignment of new technical capabilities all had favorable impacts on the overarching success and performance of SMEs. The findings also revealed that entrepreneurial orientation hardly mediates the impact of entrepreneurial orientation on achieving SMEs’ performance.

Abdulrab et al. (2020) used a quantitative research approach to conduct a research project on the general influence of entrepreneurial orientation and key strategic orientation drivers on the financial and nonfinancial performances of SMEs. The results demonstrated that entrepreneurial orientation, market orientation (MO), and technology orientation (TO) each had a constructive and substantial influence on SME financial performance and that MO and TO had a constructive and substantial adverse effect on nonfinancial performance. It was also discovered that EO seemed to have little or no negative impact on the nonfinancial performance of SMEs. According to the research conclusions, SMEs should improve their understanding of the aspects of key financial and nonfinancial economic indicators to fully comprehend them and propose consistently successful strategies.

Alsolamy (2019) investigated the practical roles of EO and innovation capacity (IC) in the long-term competitive advantage of SMEs and found that EO does indeed have a massive influence, both in terms of innovation capacity and competitive advantage. Furthermore, the final assessment of the research findings indicated that Saudi social venture enterprises’ innovativeness has a favorable influence on their long-term significant competitive advantage.

## Conceptual model

**Figure 1:**
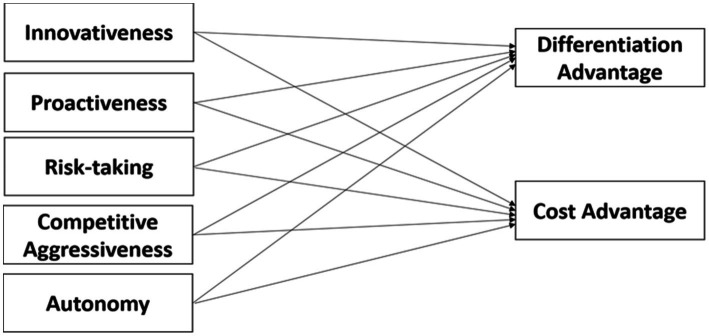
Conceptual framework.

The proposed conceptual structure of this research study, which was designed to evaluate the research question, is illustrated in Figure 1.

## Materials and methods

### Sample and procedure

Through social media platforms, including WhatsApp, LinkedIn, and email groups, a survey was made available online to Saudi SMEs in the Ha’il region. The respondents had to complete the survey in Arabic using Google Forms, which were utilized to gather the data. It took approximately 2 months to acquire all the data. Of the 231 questionnaires received, 11 were rejected due to missing or incomplete information. For additional analysis, 220 responses were recorded. A total of 109 service SMEs and 111 product SMEs participated in this study, accounting for 49.5 and 50.5% of the total participants, respectively. With regard to the participants’ work experience at their respective firms, 104 (47.3%) had less than 1 year of experience, 63 (28.6%) had 1–5 years of experience, 26 (11.8%) had 5–10 years of experience, 20 (9.1%) had 11–15 years of experience, and 7 (3.2%) had more than 15 years of experience. Similarly, 62 (28.2%) of the participants were owners, 47 (21.4%) were managers, 90 (40.9%) were owners and managers of their firms, and 21 (9.5%) were staff. In terms of the number of employees at the firm, 119 firms had fewer than 25 staff members, 56 firms had 26–49 staff members, 36 firms had 50–150 staff members, and 9 firms had 151–250 staff, accounting for 54.1, 25.5, 16.3, and 4.1% of the total participants, respectively (see Table 1).

**Table 1:** Demographic information.

Controls		Variance	
Participant’s Position	Owner Manager Owner and Manager Staff	62 47 90 21	(28.2%) (21.4%) (40.9%) (9.5%)
Year of Experience	< 1 year 1–5 years 5–10 years 11–15 years > 15 years	104 63 20 7 26	(47.3%) (28.6%) (9.1%) (3.2%) (11.8%)
Number of Employees	< 25 26–49 50–150 151–250	119 56 36 9	(54.1%) (25.5%) (16.3%) (4.1%)
SME Type	Service Product	109 111	(49.50%) (50.50%)

### Measures

#### Entrepreneurial orientation

The research examined five facets of EO, including three items pertaining to innovativeness, three items pertaining to proactiveness, three items pertaining to risk-taking, three items pertaining to competitive aggressiveness, and three items pertaining to autonomy as a sense of self-reliance. The items’ compositions were taken from Njoroge (2015) and Anwar and Shah (2020). Each item was evaluated on a Likert-type scale, with a maximum score of five.

#### Competitive advantage

Both cost advantage and differentiation advantage (consisting of four separate factors) were investigated in this study as potential components of competitive advantage (three items). In this particular study, 5-point Likert scales were utilized to quantitatively analyze several aspects of competitive advantage (Becker et al., 2022).

### Data analysis

Data were analyzed using the statistical program SmartPLS v4.0.6.9. In the first step of this process, various measurement model methodologies, such as Cronbach’s alpha (CA), extracted composite reliability (CR), heterotrait-monotrait (HTMT) ratio, and average variance (AVE), were investigated. Second, to examine the theoretical model, this study employed discriminant validity. The structural model was evaluated in the third phase by looking at the common method bias (variance inflation factor), coefficient of determination (R2), predictive relevance (Q2), and standardized root mean square residual (SRMR). Structural equation modeling (SEM) was another approach that was utilized in this investigation to examine the hypotheses.

## Statistical analysis and results

### Measurement model

In several instances, CA was utilized to obtain the reliability of the different scales. The measuring scales’ validity was determined to be substantial, with values of 0.870 for Autonomy, 0.852 for Competitive Aggressiveness, 0.834 for Innovativeness, 0.838 for Proactiveness, 0.801 for Risk-Taking, 0.768 for Cost, and 0.860 for Differentiation. The internal consistency reliability was deemed to be adequate (i.e., equal to or above 0.7, as suggested by Hair et al., 2017) in the current investigation and varied from 0.883 to 0.920. Additionally, the current investigation revealed an AVE of at least 0.50 (Fornell and Larcker, 1981; Chin, 1998; Al-Mamary, 2022a, 2022b; see Table 2).

**Table 2:** Measurement model.

Construct	Code	Loading	CA	CR	AVE
Autonomy			0.870	0.920	0.794
	Auto1	0.907			
	Auto2	0.865			
	Auto3	0.900			
Competitive aggressiveness			0.852	0.910	0.772
	ComAg1	0.905			
	ComAg2	0.843			
	ComAg3	0.887			
Innovativeness			0.834	0.900	0.751
	Innov1	0.846			
	Innov2	0.861			
	Innov3	0.892			
Proactiveness			0.838	0.903	0.756
	Proac1	0.864			
	Proac2	0.858			
	Proac3	0.886			
Risk taking			0.801	0.883	0.716
	Risk1	0.850			
	Risk2	0.819			
	Risk3	0.868			
Cost			0.769	0.866	0.684
	Cost1	0.824			
	Cost2	0.801			
	Cost3	0.856			
Differentiation			0.860	0.906	0.706
	Differ1	0.777			
	Differ2	0.853			
	Differ3	0.868			
	Differ4	0.860			

An HTMT test was carried out to determine the discriminant validity of the components (Henseler et al., 2015). The HTMT ratio must be lower than the benchmark value of 0.85 to show that discriminant validity was achieved (Table 3). The fact that none of the figures were higher than the threshold of 0.85 suggests that discriminant validity remained adequate (Henseler et al., 2015). Additionally, the correlations between constructs related to the relevant construct and the AVE square root values for each construct were compared (Fornell and Larcker, 1981). The results show that the AVE values are always higher than the correlations between them (see Table 4).

**Table 3:** HTMT.

	Auto	ComAg	Cost	Differ	Innov	Proac
ComAg	0.766					
Cost	0.734	0.802				
Differ	0.799	0.782	0.740			
Innov	0.754	0.811	0.802	0.757		
Proac	0.816	0.829	0.783	0.799	0.818	
Risk	0.844	0.820	0.843	0.829	0.836	0.807

**Table 4:** Fornell–Larcker criterion.

	Auto	ComAg	Cost	Differ	Innov	Proac	Risk
Auto	0.891						
ComAg	0.661	0.879					
Cost	0.603	0.654	0.827				
Differ	0.691	0.672	0.604	0.840			
Innov	0.644	0.686	0.646	0.644	0.866		
Proac	0.698	0.702	0.631	0.678	0.685	0.869	
Risk	0.705	0.678	0.662	0.692	0.685	0.663	0.846

### Assessment of structural model

This research used the variance inflation factor (VIF) to investigate collinearity problems and common technique bias. Because Kock (2015) and Hair et al. (2017) did not discover any values equal to or lower than 3.3, they concluded that this structural model was free of bias (see Table 5).

**Table 5:** Structured model results.

Construct	R2	Adj. R2	f2	Q^2^predict	VIF	SRMR
Cost	0.555	0.550		0.536		0.035
Differ	0.616	0.612		0.606		
Auto*Cost			0.00416		2.575	
ComAg*Cost			0.038852		2.596	
Innov*Cost			0.032943		2.509	
Proac*Cost			0.016248		2.688	
Risk*Cost			0.051372		2.637	
Auto*Differ			0.051501		2.575	
ComAg*Differ			0.030258		2.596	
Innov*Differ			0.010946		2.509	
Proac*Differ			0.030872		2.688	
Risk*Differ			0.050435		2.637	

In this particular investigation, a Harman single-factor test was also utilized to assess common technique variance (Harman, 1960). If a factor analysis shows a single-component structure or if the original single factor explains more than 50% of the variation in the observations, this suggests that the current data are sensitive to typical technique bias. However, if the original single factor explains less than 50% of the variation in the observations, this suggests that the data are not sensitive to typical technique bias.

**Figure 2:**
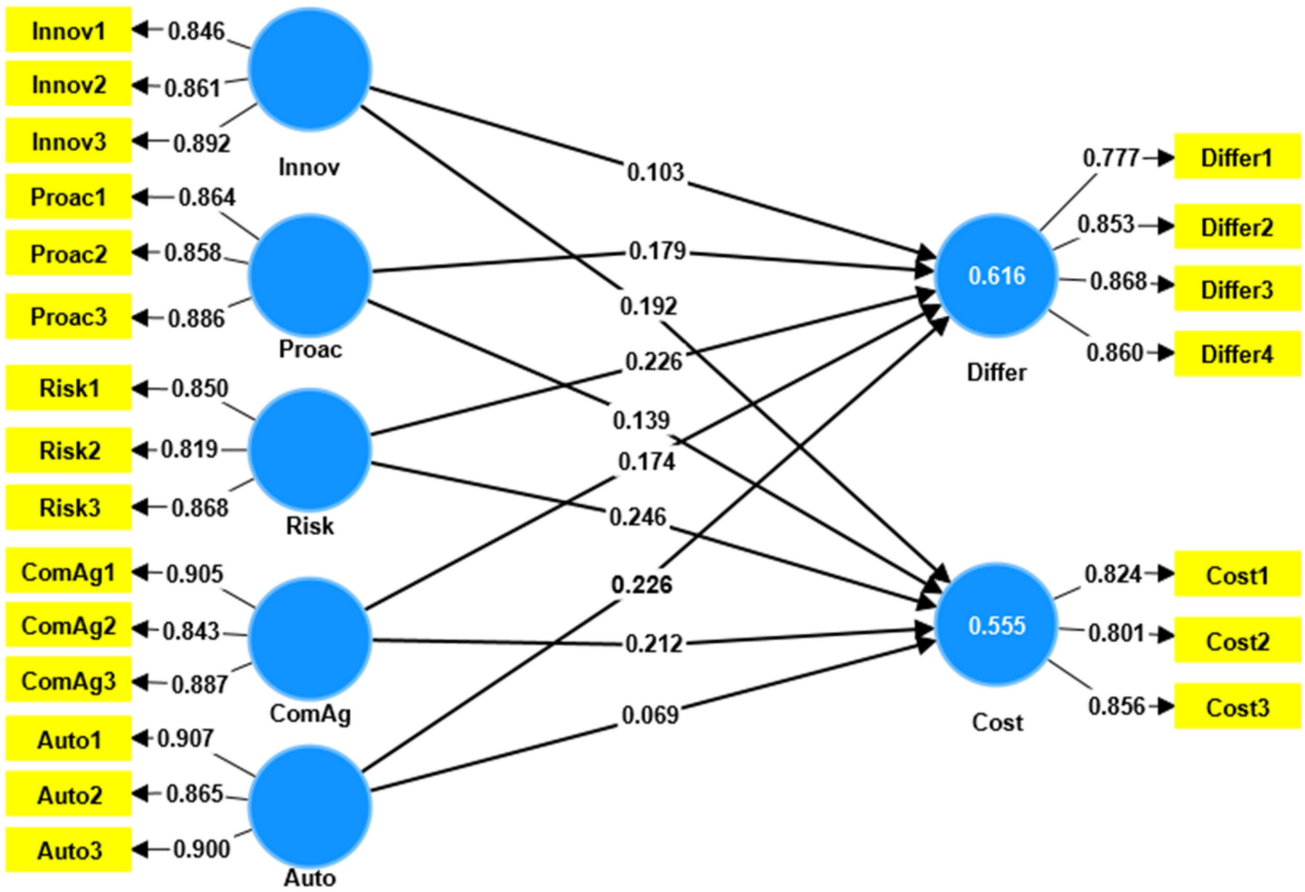
Partial least square SEM model.

Furthermore, the results showed that EO explained 55.5% of the variance in cost. EO also explained 61.6% of the variance in differentiation. According to Cohen (2013), the acquired R2 values have an adequate degree of explanatory power, which is an indication of a substantial model. While the accepted R2 rule of thumb varies, Cohen (2013) considered R2 values of 0.26 and above to be significant, indicating that the predicted model fits the data well. Within the scope of this analysis, endogenous variables showed R2 values of 0.555 and 0.616, respectively (see Table 5; Figure 2).

According to Geisser (1974) and Stone (1974), predictive relevance is a measure of a model’s ability to predict outcomes for data that are not included in the sample. According to Hair et al. (2017), an endogenous construct has moderate significance when it has a value of 0.02, medium significance when it has a value of 0.15, and significant significance when it has a value of 0.35. According to this rule, the endogenous variables in this study—cost and differentiation—showed large predictive relevance values (see Table 5).

SRMR, as suggested by Hu and Bentler (1998) was used to determine how well the data fit. The SRMR was 0.035, which was less than the required 0.08 and indicated a successful match (Henseler et al., 2016).

## Discussion

The purpose of this research was to investigate the impact of EO factors on the competitive advantage (differentiation and cost) of Saudi SMEs in the Ha’il region. It was hypothesized that differentiation (DIFFER) would be influenced by innovativeness (INNOV). The results showed that INNOV had a positive effect on DIFFER (β = 0.103, t = 2.069, *p* < 0.05; see Table 6). Thus, Hypothesis 1 is accepted. The results show that INNOV is a key predictor of differentiation strategy for the business owners of SMEs in the Ha’il region of Saudi Arabia. Entrepreneurs who think creatively produce unusual solutions that might be critical to their clients. This finding of differentiation and innovativeness confirms the findings of Lumpkin and Dess (1996a), Zeebaree and Siron (2017), and Hossain and Azmi (2020). According to the research findings, INNOV has a direct and positive impact on cost advantage (COST; β = 0.192, t = 2.992, *p* < 0.05; see Table 6). This supports Hypothesis 2. This study supported evidence from previous observations (e.g., Porter, 1985; Dowling and McGee, 1994; Zeebaree and Siron, 2017). Leutner et al. (2014) found that innovativeness had an insignificant influence on the cost advantage strategy. The findings of the current study, however, contradict those of Leutner et al. (2014). This may be explained by the fact that introducing cost-effective designs for established firms’ product/service categories necessitates some form of innovation.

**Table 6:** Hypothesis constructs.

Effects	Relations	β	Mean	SD	*t*-value	*value of p*	Decision
H1	INNOV - > DIFFER	0.103	0.106	0.050	2.069	0.039*	Supported
H2	INNOV - > COST	0.192	0.191	0.064	2.992	0.003*	Supported
H3	PROAC - > DIFFER	0.179	0.175	0.048	3.710	0.000**	Supported
H4	PROAC - > COST	0.139	0.140	0.060	2.329	0.020*	Supported
H5	RISK - > DIFFER	0.226	0.226	0.047	4.764	0.000**	Supported
H6	RISK - > COST	0.246	0.244	0.077	3.204	0.001*	Supported
H7	COMAG - > DIFFER	0.174	0.173	0.049	3.521	0.000**	Supported
H8	COMAG - > COST	0.212	0.211	0.060	3.533	0.000**	Supported
H9	AUTO - > DIFFER	0.226	0.227	0.050	4.475	0.000**	Supported
H10	AUTO - > COST	0.069	0.072	0.057	1.207	0.227	Rejected

* *p* < 0.05;

** *p* < 0.1.

The impact of proactiveness (PROAC) on DIFFER was also investigated in this study. There was a statistically significant correlation between PROAC and DIFFER (β = 0.179, t = 3.710, *p* < 0.1; see Table 6). Gitau et al. (2016) found that for small firms to be active in identifying and exploiting business opportunities, they must be proactive. These findings also confirmed the conclusions of other studies on this subject (e.g., Lilien et al., 2002; Hughes and Morgan, 2007; Zeebaree and Siron, 2017; Hossain and Azmi, 2020). However, this outcome is contrary to that of Lechner and Gudmundsson (2014) and Leutner et al. (2014), who found inconsistencies between firms’ proactiveness and differentiation strategies. PROAC was also thought to have a substantial impact on COST. PROAC was found to have a significant effect on COST (β = 0.139, t = 3.329, *p* < 0.05; see Table 6). Allen et al. (2006), Hughes and Morgan (2007), and Zeebaree and Siron (2017) all found similar findings. Contrarily, the results of the present study do not align with earlier research by Leutner et al. (2014), which revealed that proactiveness had no appreciable impact on cost advantage.

The research findings of this study have demonstrated that taking risks (RISK) has a direct and positive impact on DIFFER (β = 0.226, t = 4.764, p < 0.1; see Table 6). Thus, Hypothesis 5 was accepted. This finding is consistent with those found in earlier studies (e.g., Morris and Kuratko, 2002; Dewan et al., 2007; Leutner et al., 2014; Zeebaree and Siron, 2017; Hossain and Azmi, 2020). It was also anticipated that the level of RISK would have a significant effect on COST. According to the findings, there was a statistically significant relationship between RISK and COST (β = 0.246, t = 3.204, p < 0.05; see Table 6). It appears that these findings can be attributed to the fact that taking risks should be more significant in the case of small businesses to achieve cost leadership than they should be to achieve distinctiveness. These findings are comparable to those found in earlier studies (e.g., Leutner et al., 2014; Zeebaree and Siron, 2017).

According to the findings of this study, which can be found in Table 6 (β = 0.174; t = 3.533; p < 0.1), competitive aggression (COMAG) was discovered to have an influence on DIFFER. Therefore, Hypothesis 7 has been shown to be correct. In addition, COST was significantly affected by COMAG (β = 0.212, t = 4.620, p < 0.05; see Table 6). Therefore, the validities of Hypotheses 7 and 8 were confirmed. The findings of this study provide substantial support for the results reached by other investigations on this subject (e.g., Porter, 1985; Blumentritt and Danis, 2006; Leutner et al., 2014). According to Porter (1980), for a cost leadership strategy to be successful, a large portion of the market is required.

Furthermore, the impact of autonomy (AUTO) on DIFFER was investigated in this study. The results show that taking risks (RISK) has a significant impact on DIFFER (β = 0.226, t = 4.475, p < 0.1; see Table 6). Thus, Hypothesis 9 was supported. This finding is consistent with earlier observations (e.g., Lumpkin and Dess, 1996b; Hughes and Morgan, 2007; Lumpkin et al., 2009; Leutner et al., 2014). This could be related to the fact that when organizations empower their employees and give them more autonomy, people are more likely to be creative, come up with new ideas, engage in open communication, and be more focused on customer involvement and orientation. Finally, this study’s research findings revealed that AUTO had no effect on COST (β = 0.069, t = 1.207, p < 0.05; see Table 6). This means that Hypothesis 10 has been rejected. Thus, the outcomes conflict with previous studies (e.g., Leutner et al., 2014; Grant, 2021). Therefore, it is possible that the attributes of SMEs’ owners, managers, or employees in the Ha’il region can drive them to be empowered and creative in their businesses regardless of the cost strategy pursued.

In a nutshell, the findings of this study indicate that people who have a more entrepreneurial mindset are not all that different from businesses that have an entrepreneurial mindset, as long as both are provided with an environment that promotes their success. This is the main takeaway from the study.

### Theoretical implications

The results of this study expand the scope of EO research and demonstrate that SMEs that support and promote innovative ideas, take advantage of first-mover opportunities, and anticipate future events outperform competitors who set high market share goals or use aggressive measures, such as price cuts (Lumpkin and Dess, 1996a), to achieve competitive advantage (differentiation and cost). It is now well established that entrepreneurial inclination influences competitive advantage. However, the authors of this study did not conduct any analyses to determine the nature of the connection that exists between the EO aspects (innovation, proactivity, risk-taking, aggression in the marketplace, and autonomy) and the competitive advantage dimensions (difference and cost). This study contributes to the existing body of knowledge by investigating the effects of EO’s five aspects on the dimensions of competitive advantage held by SMEs located in the Ha’il region.

In addition, there has been a dearth of empirical evidence regarding Saudi SMEs as a consequence of the theoretical findings of earlier research on the impact of autonomy on cost advantage (e.g., Lumpkin and Dess, 1996b; Hughes and Morgan, 2007; Lumpkin et al., 2009; Leutner et al., 2014; Grant, 2021). By conducting an investigation of previous research studies’ hypotheses among Saudi SMEs in the Ha’il region, the present study contributes to the existing body of information.

### Practical and managerial implications

This study provides useful insights into the ways in which EO might help build a company’s competitive edge. It is critical to recognize that EO is the starting point for developing and implementing competitive advantage initiatives. SME owners or managers should improve their awareness and knowledge of the importance of research and development, technological leadership, proactive behaviors, and employee empowerment. Furthermore, the research provided a practical contribution by illustrating how Saudi entrepreneurs may differentiate their services through their entrepreneurial approach (EO). In addition, the findings of this research have the potential to act as a reliable reference for those who work in commercial settings. The findings indicate that EO variables are key and relevant elements in cost strategy and differentiation.

According to the findings of this study, if the owners or managers of SMEs utilize the findings by considering the structures, strategy-making processes, and business attributes that are characterized by their inventiveness, proactiveness, risk-taking, aggressiveness in competition, and autonomy, they will increase their firms’ competitive advantage. The findings may also be valuable in supporting SMEs in being successful, as SMEs are key contributors to the development and growth of the economy.

## Conclusion, limitations, and directions for future research

The research was conducted in the Ha’il region of Saudi Arabia on SMEs to see how the entrepreneurial practices of these companies affected their competitive edge. In the Ha’il context, the study placed particular emphasis on the advantages that SMEs have over larger corporations in terms of competitive advantage. These advantages include autonomy, innovativeness, risk-taking, proactivity, and aggressive competition.

Although all of this study’s objectives were accomplished, there are certain limitations that should be addressed, and relevant suggestions for future research should be made. A very small sample size calls for an extensive amount of replication. Since this study is based on cross-sectional data, further longitudinal research is needed to learn more about the problem, determine how the different parts interact with each other, and see if the results would be different if longitudinal data were used instead of cross-sectional data. Second, this study’s data were collected from 220 SMEs in Ha’il, Saudi Arabia. Therefore, future studies could expand the sample to include all other SMEs from different parts of Saudi Arabia so that the results can be used in a wider range of situations.

Third, the current study adopted a quantitative methodology and distributed questionnaires to the managers or owners of SMEs. Thus, future studies should consider obtaining more in-depth qualitative data from SMEs’ owners or managers. Future research may employ both quantitative and qualitative approaches to produce more accurate and comprehensive findings.

Fourth, the investigation of mediating factors, such as strategic orientations, Organizational Citizenship Behavior (OCB), learning orientation, and knowledge management, should be covered in future research. Links between the model’s direct and indirect paths may be examined using a variety of methods.

## Data availability statement

The raw data supporting the conclusions of this article will be made available by the authors, without undue reservation.

## Author contributions

MT: conceptualization, literature review, resources, data collection, writing—original draft preparation, and project administration. MAl: conceptualization, literature review, resources, data collection, and writing—original draft preparation. YA-M: conceptualization, methodology, software, validation, formal analysis, resources, data collection, and writing—original draft preparation. MAb: conceptualization, methodology, software, validation, formal analysis, resources, data curation, and writing—original draft preparation. All authors contributed to the article and approved the submitted version.

## Funding

This research has been funded by the Scientific Research Deanship at the University of Ha’il—Saudi Arabia, through project number RD-21 107.

## Conflict of interest

The authors declare that the research was conducted in the absence of any commercial or financial relationships that could be construed as a potential conflict of interest.

## Publisher’s note

All claims expressed in this article are solely those of the authors and do not necessarily represent those of their affiliated organizations, or those of the publisher, the editors and the reviewers. Any product that may be evaluated in this article, or claim that may be made by its manufacturer, is not guaranteed or endorsed by the publisher.
